# On the First Use of the Term Man-Midwife

**Published:** 1812-08

**Authors:** 


*To the Editors of the Medical and Physical Journal.*

GENTLEMEN,

INQUIRY was made in your 26th Vol. No. 149, p. 41, "When and by whom the compound word *Man-midwife* was first used;" which your correspondent supposed would be found to be between the years 1672 and 1705. The following extract will shew that this word was in use in 1637.

"If such slender reasons as these might prevaile," (against the publication of a Treatise on Midwifery in the vulgar tongue,) "then might there perhaps a great number perish, before ever they saw the light, who otherwise might live and increase the number of God's church by their off-springs, and perhaps also a great deale more worke might be made for *Men-midwives*, then yet is, although there be too much already, and some perhaps for private profit have too farre already incroached upon women's weaknesses and want of knowledge in these their peculiar businesses."—Translator's Preface to "The Expert Midwife, &c. compiled in Latine by the Industry of *James Rueff*, a learned and expert Chirurgion: and now translated into English for the generall good and benefit of this Nation." London, 1637.

 The

The word in the above extract is used as if it had been for some time in common acceptation; besides, therefore, correcting the supposition of your correspondent, it will serve to shew that Astruc was under a mistake when he stated that "the epocha of surgeons being employed to attend labors, does not go farther back than the first lying-in of Madam de la Valiere, in 1663;"* for here we have *men-midwives* familiarly spoken of nearly thirty years before the period which he assigns for their introduction.

There is an amusing passage in Madam Louisa Bourgeois' Instructions to her Daughter, which likewise proves that it was no uncommon practice to employ surgeons to attend labors long before the period stated by Astruc. She says, "There are now very few women who have such an affectionate regard for their midwives, as prevailed formerly, when it was the custom if a midwife died for her patients to put on deep mourning for her, and they prayed God not to send them any more children, (which indeed was not right in them, but their regard to their midwives carried them so far;) whereas now-a-days they use them as they use the work-women in the vineyards, changing them every year, and acting upon the principle of so much work so much pay. There must be a good deal of artifice to make a dose palatable to a patient easily disgusted with his medicines; and this is the case with the young women of the present age, *who even with their first children make choice of a man to attend them in their labors.* I blush for them! for it requires a great degree of effrontery to submit to that without an absolute necessity. I will venture to say, that neither their mothers nor grandmothers ever employed them; indeed we may find many women of loose character who would make a difficulty of it."†

* Astruc's Elements of Midwifery, translated by Ryley, 1766.

† Il se trouue bien peu de femmes qui affectionnent leurs sages-femmes, comme elles faisoient le temps passé, que quand les sages-femmes mouroient elles en menoient grand dueil, et prioient Dieu de ne leur plus enuoyer d'enfans, (qui n'estoit pas bien faict, mais leur affection les portoit à cela;) maintenant plusieurs s'en seruent, comme d'vne femme de vendange, ou tous les ans on change de vendangeurs, tant tenu, tant payé. Il faut bien de l'artifice à vne saulce pour la faire trouuer bonne, à vn malade bien degousté; *comme sont nos ieunes femmes, qui dés leurs premiers Enfans, font eslection d'vn homme pour les accoucher*, i'en rougis pour elles! car c'est vne effronterie trop grande que se resoudre à cela sans besoin, je m'asseure que leur mere, ny grand'mere ne s'en sont pas seruies: il se trouuera des femmes de mauuaise vie qui en feroient de la difficulté.—*Observations diuerses sur la sterilité, perte de fruict, &c. par L. Bourgeois dite Boursier sage Femme de la Roine. Paris,* 1642.

It is somewhat extraordinary that Astruc should have fixed the epocha of the first introduction of men into the practice of midwifery so late as 1663, since he might have learned from the above book of Madame Bourgeois, which he acknowledges to have read, and from some other books, that it was becoming customary to employ accoucheurs much earlier; it is indeed impossible to suppose, as Astruc seems to have imagined, that the employment of men in such cases arose from a fashion set by princesses and queens, or that it was adopted on a sudden. It must have been growing into use for a considerable space of time, the women being driven to it by the ignorance and unskilfulness of the midwives. It is probable that Julian Clement, whom Astruc speaks of as the first regular accoucheur, had a good deal of experience in midwifery, or Louis XIV. would hardly have entrusted to him the care of Madame de la Valiere, his favorite mistress, during her first lying-in.

*Half-moon-street, June* 5, 1812. S. M.

